# A Communication-Efficient Secure Routing Protocol for IoT Networks †

**DOI:** 10.3390/s22197503

**Published:** 2022-10-03

**Authors:** Yuma Shibasaki, Keiichi Iwamura, Koya Sato

**Affiliations:** 1 Department of Electrical Engineering, Tokyo University of Science, Tokyo 125-8585, Japan; 2 Artificial Intelligence eXploration Research Center, The University of Electro-Communications, Tokyo 182-8585, Japan

**Keywords:** Internet of Things (IoT), communication efficiency, secure routing, authentication

## Abstract

This paper proposes a secure routing protocol based on an ad hoc on-demand distance vector to simultaneously achieve communication efficiency and security. Many studies have discussed secure protocols. However, conventional protocols tend to exhibit low communication efficiencies owing to the long packets required by digital signatures, specifically in large-scale networks. Hence, our proposed method aims to allow the intermediate node to initiate a route reply (RREP), which is prohibited in conventional protocols because of digital signature restrictions. Based on an ID-based signature, the proposed protocol allows each intermediate node to hold a packet received from a specific node in the past. Each node then appends it to the route request of another node and generates its own signed RREP. This procedure guarantees that a third party holds the route to the destination. Theoretical evaluations demonstrate that the proposed method outperforms the communication efficiency of conventional secure protocols. We measured the time required for routing (i.e., the sum of communication and cryptographic calculation times) using a Raspberry Pi with C language. We show that the proposed protocol can improve the average routing time by more than 3× compared with conventional methods when 30 relay nodes are randomly distributed in a 300-square meter area.

## 1. Introduction

Broad demand for the Internet of Things (IoT) has led to an increase in wireless devices and their applications over the last decade. In 2020, the number of mobile devices worldwide exceeded 10 billion, which is expected to increase with the further application of such networks [1]. Decentralized wireless communications based on mobile ad hoc networks (MANETs) are recognized as the core communication technologies for realizing large-scale IoT systems for applications such as agricultural monitoring [2] and healthcare management [3]. MANETs enable wireless devices to communicate with each other without a base station (BS).

Although this field has been widely discussed for more than 20 years, novel wireless applications (including IoT systems, disaster networks [4], autonomous vehicle networks, unmanned aerial vehicle (UAV) networks [5], and decentralized federated learning (DFL) [6]) have increased the importance of MANETs. For example, federated learning (FL) [6] enables machine learning to be performed over distributed networks without any data disclosure from edge devices. Specifically, its fully decentralized form, called decentralized FL (DFL), allows data analysis over decentralized networks, which can be realized using MANET-based communications. FL/DFL is expected to be a privacy-preserving learning scheme; however, it should be performed with high security and communication efficiency. Furthermore, information gathering based on the Age of Information (AoI) has been recently discussed. The AoI is an information-theoretic criterion expressing the freshness of the information of interest [7]. Communication design based on AoI enables IoT networks to collect massive amounts of sensing information with low latency. As previously discussed, recent trends in IoT show the requirements for low-latency communications in large-scale networks.

In MANETs, nodes must construct a route from the source to the destination nodes in advance. However, typical routing protocols (e.g., ad hoc on-demand distance vector (AODV) and dynamic source routing (DSR)) allow any node to freely join the network. Thus, these protocols are vulnerable to cyber-attacks from malicious nodes such as communication interceptions, route falsifications, and black hole attacks [8]. Several of the above-mentioned applications of MANETs manage privacy and safety information; therefore, in addition to the communication issue, the security of MANETs is a key topic in the future IoT systems.

Various studies have proposed secure routing protocols to improve security issues in MANETs [9,10,11,12,13,14,15,16,17,18]. The different types of secure routing protocols can be roughly divided into (a) cryptography-based approaches and (b) trust-based approaches. In general, cryptography-based routing protocols require nodes to append a digital signature to the routing packet in order to guarantee the validity of the packet and prevent forgery and falsification [9,10,11]. A digital signature can deal with attacks. However, it degrades communication efficiency because cryptographic information is added to the relayed packet hop-by-hop. This feature leads to an increase in communication overhead and a low packet delivery rate (PDR). Furthermore, due to the limitations of packet authentication, related methods must perform end-to-end communication whenever necessary to ensure the integrity of routing packets. This feature tends to increase the number of hops as the network size increases, as intermediate nodes are prohibited from initiating the route reply. Most wireless devices in MANETs have limited computational capabilities and communicate using finite wireless resources. Therefore, a secure routing protocol should work with a limited increase in the routing packet size.

In contrast, trust-based protocols [16,17,18] can prevent malicious nodes from joining networks while preserving both computational and communication efficiencies by introducing a trust value based on the past behavior of the target node. However, most trust-based methods allow malicious nodes to attack trust, such as forgery of trust [19]; that is, these techniques may lack security performance compared to cryptography-based methods.

Motivated by this background, we propose a cryptography-based routing protocol that achieves both communication efficiency and security performance. In our proposed protocol, while digital signatures validate the route information created by each node, the intermediate nodes in the route save previously received packets from the destination node. Thus, they can prove that there is a valid route to the destination by disclosing these packets to other nodes. Thus, intermediate nodes are encouraged to begin constructing a route by exchanging route information with a source node while ensuring safety and security. The protocol is based on an ID-based signature scheme. The ID-based signature allows nodes to sign packets based on their secret keys and verify their integrities [20]. Unlike public key-based signatures, this feature removes the requirement for public key infrastructure (PKI), helping to prevent impersonation attacks.

The major contributions of this study are as follows:

- We propose a routing scheme that can reduce the number of hops in the routing phase, as with non-secured AODV, while maintaining security performance.
- We analyze the practical overhead routing performance, which comprises the sum of the computation and communication times. Emulation analyses based on an IoT device reveal that the proposed method can improve overhead performance compared with related protocols, even in a small-scale network.

Note that this work is an extended version of our earlier work in [21]. In the present work, we compare the performance of our proposed approach with a novel related method proposed in [11], providing implementation results with a Raspberry Pi. These results reveal that the protocol proposed in this paper improves the routing efficiency.

The remainder of this paper is organized as follows. In Section 2, we summarize related works. Section 3 defines the system model, and we present the proposed routing protocol in Section 4. A comparison of different methods is presented in Section 5, and the performances of the proposed and the comparison methods are evaluated in Section 6. Finally, we conclude the paper in Section 7.

## 2. Related Works

There has been a wide range of discussions on routing over the last 20 years. Although MANETs have attracted considerable attention, recent demands for IoT applications have raised practical implementation issues from various viewpoints, such as energy efficiency [22,23], communications efficiency [24], and security [14]. In this section, we first review the recent research trends related to secure routing protocols. Secure routing protocols can be divided into cryptographic-based methods [9,10,11,12,14,15] and trust-based methods [16,17,18]. We summarize these approaches here along with their limitations and differences.

Trust-based methods do not require cryptographic processing, thereby preventing illegal nodes from joining the route while achieving low computational complexity. Anjail et al. proposed a trust-based method based on the DSR protocol that prevents packet drops by sharing attacker information within a network [16]. Jusik et al. introduced a base station to calculate the trust value to avoid trust tampering at the nodes [17]. Furthermore, Ref. [18] proposed a protocol that considers resource consumption at nodes. By considering network conditions (such as topology, node power consumption, and channel states), this method is able to identify whether a communications failure is due to an actual failure or a node error. Subsequently, if communications are excessively concentrated on a node, an alternative route is selected. However, detecting a trust forgery is difficult when there are many attackers in the network; thus, the security performance of trust-based routing degrades significantly.

By contrast, a cryptographic-based secure protocol can successfully prevent forgery and tampering with route packets via computational security. Adil et al. proposed a method for secure node authentication by encrypting the medium access control (MAC) address and sending it to a BS [14]. However, a malicious node can join a network via authentication tampering. Black hole attacks can be prevented by using digital signatures to prevent forging and falsification of messages. Zapata et al. proposed Secure-AODV (SAODV), incorporating digital signatures and hash chains into AODV to prevent black hole attacks [25]. Authenticated routing for ad hoc networks (ARAN) [10] adds the digital signature and ID of the intermediate nodes to the routing packet to prove that the relay node correctly sent the message. These cryptographic-based methods are theoretically resistant to packet forgeries and tampering. However, because these protocols allow the nodes to append signature information to the relay packet, communication efficiency tends to decrease hop-by-hop. Motivated by this background, Ref. [11] proposed an efficient and secure routing protocol that integrates multiple signatures into a single size by incorporating the aggregate signature into the DSR. This method can reduce the packet length. However, this method (and the related methods presented above) prohibits the intermediate nodes from generating a route reply (RREP) packet to secure the route; thus, the communication efficiency becomes degraded in large-scale networks incorporating IoT systems.

To the best of our knowledge, there is currently no routing method that achieves both high communication efficiency and security. Therefore, we propose a cryptographic-based routing protocol that achieves both communication efficiency and security performance.

## 3. System Model

**Figure 1:**
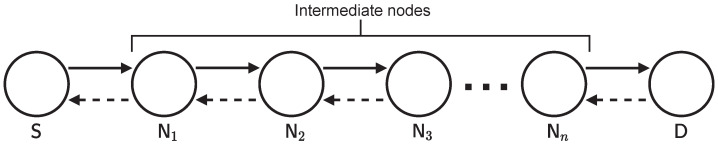
Network model.

We first describe the network configuration, security requirements, and attacker model. This study considers a situation in which the source node S constructs a route to the destination node D, as shown in Figure 1. Assuming *n* intermediate nodes between S and D, each intermediate node can be denoted by $N_{i}\left(i=1,2,...,n\right)$. We assume that the nodes use digital signatures based on an ID-based signature. To this end, a server issues a secret key and unique ID to generate a digital signature for each node in advance. An ID-based signature enables the nodes to sign packets based on their secret keys and verify them based on the signers’ IDs in the packets. This feature removes the requirement for any centralized server, including public key infrastructure (PKI), unlike public key-based methods [20]. Subsequently, the nodes construct a route in a fully distributed manner. Furthermore, it is assumed that all nodes employ a tamper-resistant global positioning system (GPS) to append their location information to the routing packets.

Based on the security requirements in [10,26], we set the following requirements in our proposed method:

- Attackers cannot spoof other nodes;
- Attackers cannot forge route information;
- Only authorized nodes can join the network, preventing packet injection attacks [27];
- The routing protocol can mitigate the effects of packet dropping and delaying attacks;
- The node that generates the RREP on behalf of the destination node can prove to a third party that the route from this node to the destination correctly exists.

Note that unlike the other methods, the proposed method is characterized by an intermediate node that can generate the RREP. Thus, we set a new fifth condition to guarantee the validity of the intermediate node. The proposed protocol is designed based on the aforementioned requirements.

## 4. Proposed Method

This section describes the proposed routing protocol.

### 4.1. Overview

The proposed method aims to enable intermediate nodes to generate RREPs as responders in AODV-based secure routing protocols to improve communication efficiency. Intuitively, this objective can be achieved through a procedure in which the node proves to a third party that the specified node has communicated with the destination node in the past. To this end, relaying nodes working with the proposed method record the routing packet generated by the destination in the past along with the routing table. In addition, each relaying node signs the relaying packet using a digital signature, and this packet is used to prove the route validity.

Furthermore, to prevent the falsification of packets and attacks by malicious nodes, our proposed method allows the source and latest intermediate nodes to add their digital signatures to the relay packet. This signature scheme uses an ID-based signature that does not require a public key certificate.

In the original AODV, a relaying packet contains hop count information, and each relay node increases this value when relaying it. However, a malicious node can falsify the hop count, which may be a clue to a black hole attack. Therefore, the relaying packet in the proposed method does not include any updated (without digital signatures) hop-by-hop information. Instead of the hop count, the packet includes the location of the destination obtained via GPS. Each node calculates the distance to the destination by comparing its location and GPS information.

The proposed method must consider (i) a route request (RREQ), (ii) an RREP phase when the destination generates the RREP (i.e., routing over end-to-end), and (iii) an RREP phase when an intermediate node generates the RREP. We describe each protocol in detail below.

### 4.2. Route Request Phase

Based on the network model illustrated in Figure 1, we describe the RREQ phase below:

1. The source node S generates an RREQ with its own signature (${\mathrm{Sig}}_{S}$) and broadcasts it to the neighboring nodes. If relay node $N_{1}$ receives this packet, this phase can be expressed as
  (1) $$S\to N_{1}:{\left(\mathrm{RREQ}\right)}_{{\mathrm{Sig}}_{S}}$$
  For this RREQ, the packet valid time (LT) and its own location information (${\mathrm{LI}}_{S}$) are added to the RREQ of the original AODV (${\mathrm{RREQ}}_{\mathrm{AODV}}$). However, information on hop counts is *not* included, as the digital signature-aided routing prohibits any nodes from updating the hop count. Based on ${\mathrm{LI}}_{S}$, the intermediate nodes estimate the distance from the source node, and can stop the routing if far from the source. Thus, the contents of the RREQ in this method are expressed as
  (2) $$\mathrm{RREQ}=({\mathrm{RREQ}}_{\mathrm{AODV}},\;\mathrm{LT},\;{\mathrm{LI}}_{S})$$
2. Node $N_{1}$ verifies the signature of node S in the received packet, establishes a path in the direction of S, and stores the routing information and sent RREQ in its own table. Subsequently, $N_{1}$ adds its own signature and address (${\mathrm{ID}}_{N_{1}}$) to the RREQ and broadcasts it. When this packet reaches relay node $N_{2}$, this phase can be written as
  (3) $$N_{1}\to N_{2}:{\left({\left(\mathrm{RREQ}\right)}_{{\mathrm{Sig}}_{S}},\;{\mathrm{ID}}_{N_{1}}\right)}_{{\mathrm{Sig}}_{N_{1}}}$$
3. Upon receiving the RREQ from $N_{1}$, $N_{2}$ verifies the signatures of S and $N_{1}$, establishes a path in the direction of S, and stores the RREQ from $N_{1}$. The signature and address of $N_{1}$ are replaced with the signature and address of $N_{2}$.
4. The storing and broadcasting processes are repeated until this RREQ reaches D or an intermediate node that knows a valid route to the destination node D.

Intermediate nodes do not relay the RREQ when the received time exceeds LT or the distance between their own location and ${\mathrm{LI}}_{S}$ exceeds a certain distance (e.g., 1 km).

### 4.3. Route Reply Phase-1

In the proposed method, the RREP procedure of the route is divided into two situations: (i) when D generates the RREP, and (ii) when the intermediate nodes on the route generate the RREP instead of D. First, we describe the former case.

1. D generates an RREP signed with its own signature and broadcasts it. If $N_{n}$ receives this packet, this phase can be denoted by
  (4) $$D\to N_{n}:{\left(\mathrm{RREP}\right)}_{{\mathrm{Sig}}_{D}}$$
  This RREP now employs the destination’s location information (${\mathrm{LI}}_{D}$) compared with the RREP of the original AODV (${\mathrm{RREP}}_{\mathrm{AODV}}$), while information about the hop count is excluded. The contents of the RREP in this method are expressed as follows:
  (5) $$\mathrm{RREP}=({\mathrm{RREP}}_{\mathrm{AODV}},\;\mathrm{LT},\;{\mathrm{LI}}_{D})$$
2. $N_{n}$ verifies the signature from D in the received packet and stores the routing information and sent RREP in its own table. Subsequently, $N_{n}$ adds its own signature and address (${\mathrm{ID}}_{N_{n}}$) to the RREP and forwards it. This is denoted by
  (6) $$N_{n}\to N_{n-1}:{\left({\left(\mathrm{RREP}\right)}_{{\mathrm{Sig}}_{D}},\;{\mathrm{ID}}_{N_{n}}\right)}_{{\mathrm{Sig}}_{N_{n}}}$$
3. $N_{n-1}$ receives the RREP from $N_{n}$, verifies the signatures of D and $N_{n}$, establishes a path in the direction of D, and stores the RREP from $N_{n}$. Subsequently, $N_{n-1}$ replaces the signatures and addresses of $N_{n}$ with its own signature.
4. This RREP storage and forwarding procedure is repeated until the RREP reaches S.

As in the RREQ phase, this relaying is stopped when the distance between a node and ${\mathrm{LI}}_{D}$ exceeds a threshold.

### 4.4. Route Reply Phase-2

Next, we show an additional case, in which intermediate nodes on the route generate RREPs instead of D.

1. When the intermediate nodes $N_{r}$ receive an RREQ from S and generate an RREP instead of D, $N_{r}$ adds the RREP generated by itself to the RREP that it has received from D in the past (defined below as ${\left(D^{\prime }s\;\mathrm{RREP}\right)}_{{\mathrm{Sig}}_{D}}$). This can be derived by
  (7) $$N_{r}\to N_{r-1}:{\left(\mathrm{RREPR},{\left(D^{\prime }s\;\mathrm{RREP}\right)}_{{\mathrm{Sig}}_{D}}\right)}_{{\mathrm{Sig}}_{N_{r}}}$$
  where
  (8) $$\mathrm{RREPR}=({\mathrm{RREP}}_{\mathrm{AODV}},\;\mathrm{LT},\;{\mathrm{LI}}_{r}).$$
  ${\mathrm{LI}}_{r}$ is the location of $N_{r}$.
2. When $N_{r-1}$ receives the RREP defined in Equation (7), this node verifies the signatures of the responders $N_{r}$ and D in the packet. Subsequently, the RREP is forwarded along the route to S. At this time, $N_{r-1}$ adds its own signature and address to the received packet. The contents of the RREP at this time are provided by
  (9) $$\begin{array}{cc} N_{r-1}\to N_{r-2} & :{\left({\left(\mathrm{RREPR},{\left(D^{\prime }s\;\mathrm{RREP}\right)}_{{\mathrm{Sig}}_{D}}\right)}_{{\mathrm{Sig}}_{N_{r}}},{\mathrm{ID}}_{N_{r-1}}\right)}_{{\mathrm{Sig}}_{N_{r-1}}} \end{array}$$
3. $N_{r-2}$ verifies the signatures of the responder $N_{r}$, D, and the latest intermediate node $N_{r-1}$ in the packet. Subsequently, $N_{r-2}$ discards the ID and signature of $N_{r-1}$ from the received RREP and adds its own ID and signature to the packet in their place. The packet is forwarded, and this forwarding process continues until the RREP reaches S and a route to D is established.

The relaying procedure is stopped when the distance between a node and ${\mathrm{LI}}_{r}$ exceeds a threshold, which is similar to the RREQ phase.

### 4.5. Route Selection at the Source Node

When many nodes exist, the source node S may receive multiple RREPs by various routes. The source node must select the route for later communication. In this case, the source selects the packet with the highest sequence number for the routing packet generated by the destination itself. Here, even when the relay node generates the RREP, it is the sequence number in the packet generated by the destination node itself in the past that is referred to, not the sequence number in the RREP generated by the relay node.

### 4.6. Route Maintenance Phase

The constructed routes must be maintained periodically, as the communication quality of the routes may fluctuate over time based on various factors, such as multipath fading, interference, and attacks involving packet dropping or delaying. Thus, we set up an optional packet for route maintenance.

Consider a situation where node $N_{i}$ on the route detects that a node on the route cannot connect and communicate with the destination node D. In this situation, $N_{i}$ generates a route error message (RERR) and sends it to the upstream node. The format of the RERR in the proposed method is expressed as follows:

(10) $${\mathrm{RERR}}_{N_{i}}={\left({\mathrm{RERR}}_{\mathrm{AODV}},\;{\mathrm{ID}}_{N_{i+1}},\;\mathrm{LT}\right)}_{{\mathrm{Sig}}_{N_{i}}}$$

The address of the destination node D of the disabled route is written to the RERR, and each node that receives the RERR discards the route to that destination from its table. Subsequently, the node relays the RERR to its upstream node. In addition, the signature of the source of the RERR is added to the packet and verified by the receiver. To prevent a specific RERR from flowing to the network, the packet has a valid time; a node that receives a packet for which the valid time has expired discards the packet and does not forward it. In addition, the detection of route abnormalities is performed based on the packet transmission rate (PSR) using an acknowledgment packet (ACK), as in [18].

After the route is established, an ACK is sent back to the source when the destination receives an information packet from the source. Each node then calculates whether the ratio of the number of ACKs actually received to the number of messages sent is below a threshold $r_{\mathrm{ACK}}$. The packet transmission rate is calculated for each fixed number of transmitted messages *M*, that is, if $\frac{N_{\mathrm{ACK}}}{M}<r_{\mathrm{ACK}}$ (where $N_{\mathrm{ACK}}$ is the number of received ACKs), then the node generates the RERR.

## 5. Comparison Methods

In this section, we summarize three related protocols for secure routing, namely, SAODV [9], ARAN [10], and ISDSR+ [11]. A comparison of their performance with that of the proposed method is presented in Section 6.

### 5.1. SAODV [9]

This protocol utilizes a digital signature to provide non-repudiation of the non-mutable data in the route information (e.g., sequence numbers) and protect its integrity; and hash chains are used to authenticate the hop count information (HC). The protocol is summarized below.

Let us consider a case in which S attempts to establish a route to D. In this protocol, S broadcasts an RREQ packet. At this point, S determines the maximum number of hops to D (MHC), and if the RREQ does not reach D within the MHC, the RREQ is discarded. The following describes the calculation procedure implemented by the hash chains.

Node S generates a random number (seed) and stores its value in a field called Hash. The seed is input to the hash function *h*MHC times, the output is obtained, and the result is expressed as $\mathrm{TopHash}=h^{\mathrm{MHC}}\left(\mathrm{seed}\right)$. This calculation result is stored in the TopHash field as defined in the RREQ. Subsequently, S generates the RREQ by signing with its own private key. Before the generated RREQ is broadcast to the neighboring nodes, node S inputs the seed, which is generated in advance, to *h*. At this time, Hash=h(seed) is stored in the hash field.

The node that receives the RREQ verifies the attached signature using S’s public key and verifies whether the number of hops is correct by applying the hash function. As a verification procedure using the hash function, the hash value contained in the RREQ is input to the hash function exactly the number of times equal to the difference between MHC and HC, which is contained in the RREQ as TopHash. This ensures that the same value has been compared and verified. This procedure can be written as

(11) $$\mathrm{TopHash}==h^{\left(\mathrm{MHC}-\mathrm{HC}\right)}\left(\mathrm{Hash}\right),$$

where a==b indicates that a node is attempting to verify whether a=b.

After these verification processes and the establishment of a reverse route to S as AODV, the RREQ is forwarded to the destination-directed neighbor node. The node that receives this RREQ verifies the signature and calculates relevant hash chains in the same manner by repeating these procedures until the RREQ reaches D.

When D receives the RREQ and generates the RREP as an RREQ, the signature can protect the integrity and provide non-repudiation of the non-mutable data in the route information. Furthermore, hash chains with a one-way hash function can authenticate the hop count. Each node updates its routing table when the signature and verification of the RREP by the hash chain are completed. After establishing a route to the destination node, it can then forward the RREP to S. When the RREP reaches S and signature and hash chain verification are complete, a route from the source node S to the destination node D is conclusively established.

### 5.2. ARAN [10]

Similar to SAODV, ARAN uses digital signatures. However, this protocol assumes that only nodes issued with a public key certificate [28] from a certificate authority server can join the network. By requesting a public key certificate when exchanging route information, ARAN guarantees that nodes exist on the route.

This method sets a trusted certificate authority server T. T issues and distributes public key certificates to each node. The public key certificate that node A receives from T is expressed as

(12) $${\mathrm{cert}}_{A}=\left({\mathrm{IP}}_{A},K_{A+},t,e\right)K_{T-},$$

where ${\mathrm{IP}}_{A}$ is the IP address of node A, $K_{A+}$ is A’s public key, *t* is a timestamp indicating the time at which the certificate was issued by T, and *e* is the certificate validity time. The certificate is distributed by A and signed by T’s private key. When source node S establishes a route to destination node D, S generates and broadcasts an RREQ packet to its neighboring nodes. This packet can be written as

(13) $$\left(\mathrm{RDP},{\mathrm{IP}}_{D},{\mathrm{Nonce}}_{S}\right)K_{S-},{\mathrm{cert}}_{S},$$

where RDP is a packet identifier, ${\mathrm{IP}}_{D}$ is the IP address of the destination node D, and ${\mathrm{Nonce}}_{S}$ is a nonce. This RREQ packet contains a signature with S’s private key and a public key certificate issued by T. When this packet is received by S’s neighbor node B, B first verifies the signature using S’s public key and then confirms that the certificate falls within the valid time. If both are verified, then B establishes a reverse route to S. Then, B forwards the received RDP to deliver it to the destination node D. At this time, B’s signature and B’s certificate are conveyed to the received packet as

(14) $$\left(\left(\mathrm{RDP},{\mathrm{IP}}_{D},{\mathrm{Nonce}}_{S}\right)K_{S-}\right)K_{B-},{\mathrm{cert}}_{S},{\mathrm{cert}}_{B}.$$

When this packet is received by node C, which is a neighbor of B, the signatures of S and B are verified. If both are successful, C establishes a reverse path toward S, then, in addition to B, forwards the RDP itself, thereby removing B’s signature and certificate from the packet and using its own signature and certificate instead. This process is repeated until the RDP reaches D. After receiving the RDP, D verifies the signatures of S and the neighboring node, generates an RREP packet (denoted as REP in ARAN), and sends it back to C using the reverse route established during the RDP exchange. The REP generated by D is expressed as

(15) $$\left(\mathrm{REP},{\mathrm{IP}}_{S},{\mathrm{Nonce}}_{S}\right)K_{D-},{\mathrm{cert}}_{D},$$

where REP is a packet identifier, ${\mathrm{IP}}_{S}$ represents the IP address of the source node S, and ${\mathrm{Nonce}}_{S}$ is the nonce described in the RDP received from S. This REP is forwarded until it reaches node S, and the intermediate node writes the signature and verifies the signature generated by the destination. If S successfully receives the REP and verifies D’s signature and certificate, a path from S to D is successfully established.

### 5.3. ISDSR+ [11]

This protocol attempts to reduce packet length in secure routing using a signature scheme called identity-based sequential aggregate signatures (IBSAS) [29], which is an ID-based aggregate signature.

The digital signatures used in this method are based on an ID-based signature [20]. When the source node S establishes a route to the destination node D using this method, S broadcasts the RREQ to its neighboring nodes. The RREQ is allocated a signature by S. When node A, a neighbor node of S, receives the RREQ, A adds its address to the packet, generates a new signature which is an aggregate signature with S, adds the newly generated signature instead of the signature attached to the RREQ from S, and adds itself before forwarding the entire packet. This process is repeated until the RREQ reaches the destination node D. When D receives the RREQ, D verifies the aggregated signature. Subsequently, a signed RREP is generated by D and sent back to S. As in the RREQ phase, each relay node adds its address and aggregate signature to the packet and forwards the RREP until it reaches S. When S receives the RREP and verifies the aggregate signature, a path is established.

## 6. Performance Evaluation

In this section, we present the respective performance of the proposed and compared methods. We first discuss their security performance. Then, the communication efficiency performance is analyzed in terms of (a) the hop count during route construction and (b) the total byte flow during route construction. Finally, to evaluate the route construction performance of the proposed and compared IoT networks, we perform an emulation-based analysis using a Raspberry Pi.

The discussion in Section 6.2, Section 6.3, Section 6.4 follow the network model shown in Figure 1, that is, we consider a situation where *n* nodes exist between the source and destination nodes.

### 6.1. Security Performance

As defined in Section 3, we employ several requirements for the routing process. In the proposed method, all routing information packets are signed by the node that generates the packet, which prevents attackers from impersonating other nodes and can detect attacks such as falsification or forgery of packets via signature verification. Therefore, the proposed method satisfies the first and second requirements defined in Section 3 (i.e., spoofing and falsification). Furthermore, the proposed method assumes that a unique ID and the paired secret key are distributed to each node by a server in advance to utilize the ID-based signature. This feature implies that only pre-authorized nodes can join the network, thereby preventing attacks from unknown nodes such as packet injection attacks. Finally, based on the RERR, the proposed method can mitigate the effects of packet-dropping or delaying attacks by discarding low-quality communication routes.

All of the compared methods satisfy these requirements. However, they cannot satisfy the fifth requirement (i.e., that the intermediate node that generates the RREP on behalf of the destination node cannot prove to a third party that the route from this node to the destination exists and is correct). Instead, SAODV determines an option wherein the relay node generates an RREP using another signature called a *double signature*, which is received from another node. However, an attacker may continue to use a signature received in the past, even if the attacker does not hold the route to the destination, implying that the attacker can generate an RREP illegally.

In contrast, with the proposed protocol, the intermediate node can securely generate an RREP because it discloses the RREP packet that was received in the past from the destination node. In this way, the routing efficiency can be significantly improved while maintaining the security performance.

### 6.2. Minimum Number of Hops at Route Construction

For our comparison of communication efficiency, we discuss the minimum number of hops required for route construction.

The compared methods prohibit any intermediate nodes from creating an RREP, as these methods require a signature from the destination node. Thus, in all three methods, an RREQ and RREP must always begin from the end nodes. Therefore, the compared methods require 2(n+1) hops for any route construction.

**Figure 2:**
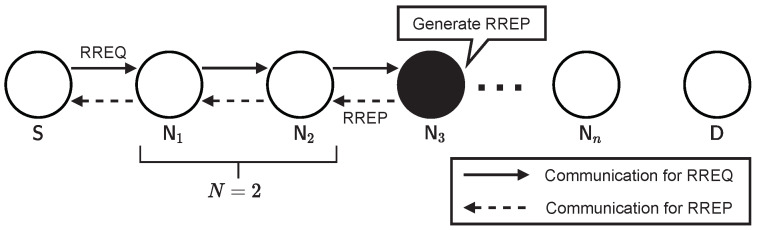
Example of the network configuration for performance evaluation (where N=2).

However, in the proposed method, if an intermediate node has an existing route to the destination, it can behave as a responder instead of a destination node. Herein, we define the number of intermediate nodes between the source node and responder as *N* (0≤N≤n−1), an example of which is illustrated in Figure 2. In the proposed method, the number of hops for route construction can be expressed as 2(N+1), implying that, in the best case, the route can be constructed with two hops for any *n*. Thus, the proposed method can significantly reduce the number of redundant hops required for route construction.

### 6.3. Total Byte Performance

In both the proposed and compared methods, the packets in the RREQ and its replies are updated hop-by-hop based on cryptographic processing. This feature changes the packet length depending on the network size. As packet length directly affects communication efficiency, we evaluate the total number of bytes in the packets flowing through the network when establishing a route.

First, the total number of bytes in AODV and DSR can be expressed as [26,30]

(16) $$\begin{array}{cc} B_{\mathrm{AODV}} & =\left(n+1\right)\times \mathrm{Size}\left({\mathrm{RREQ}}_{\mathrm{AODV}}\right)+\left(n+1\right)\times \mathrm{Size}\left({\mathrm{RREP}}_{\mathrm{AODV}}\right) \end{array}$$

(17) $$\begin{array}{cc} B_{\mathrm{DSR}} & =\left(n+1\right)\times \mathrm{Size}\left({\mathrm{RREQ}}_{\mathrm{DSR}}\right)+\left(n+1\right)\times \mathrm{Size}\left({\mathrm{RREP}}_{\mathrm{DSR}}\right), \end{array}$$

where Size(a) (bytes) is the number of bytes in a packet *a*. Furthermore, the total packet size of the SAODV can be expressed as [26]

(18) $$\begin{array}{cc} B_{\mathrm{SAODV}} & =B_{\mathrm{AODV}}+2\left(n+1\right)\times \mathrm{Size}\left({\mathrm{IDB}}_{\mathrm{Sig}}\right)+2\left(n+1\right)\times \mathrm{Size}\left(\mathrm{Hash}\right), \end{array}$$

where ${\mathrm{IDB}}_{\mathrm{Sig}}$ denotes the ID-based signature. The total packet size of ARAN can be defined as [30]

(19) $$\begin{array}{cc} B_{\mathrm{ARAN}} & =B_{\mathrm{AODV}}+2\left(2n+1\right)\times \mathrm{Size}\left({\mathrm{IDB}}_{\mathrm{Sig}}\right). \end{array}$$

The total packet size of ISDSR+ can be derived by [26]

(20) $$B_{\mathrm{ISDSR+}}=B_{\mathrm{DSR}}+2\left(n+1\right)\times \mathrm{Size}\left({\mathrm{IBSAS}}_{\mathrm{Sig}}\right).$$

where ${\mathrm{IBSAS}}_{\mathrm{Sig}}$ is the digital signature based on the IBSAS.

The packet format in the proposed method depends on whether the RREP is generated by an intermediate or destination node. Therefore, we evaluate the performance of each separately. When the destination node constructs an end-to-end route as part of the proposed method, it is expressed as

(21) $$\begin{array}{cc} B_{\mathrm{DR}}=\; & B_{\mathrm{AODV}}-2\left(n+1\right)\times \mathrm{Size}\left(\mathrm{HC}\right)+2\left(n+1\right)\times \mathrm{Size}\left(\mathrm{LT}\right)+2\left(n+1\right)\times \mathrm{Size}\left(\mathrm{LI}\right)\\ \; & +2\left(2n+1\right)\times \mathrm{Size}\left({\mathrm{IDB}}_{\mathrm{Sig}}\right)+2n\times \mathrm{Size}\left(\mathrm{ID}\right). \end{array}$$

Next, we consider the total number of bytes when the intermediate nodes generate RREPs. This packet contains the RREP that the responder node previously received from the destination node D. First, the size of the RREP generated by the destination node D is calculated as follows:

(22) $$\begin{array}{cc} \; & L_{\mathrm{DR}}=\mathrm{Size}\left({\mathrm{RREP}}_{\mathrm{AODV}}\right)-\mathrm{Size}\left(\mathrm{HC}\right)+\mathrm{Size}\left(\mathrm{LI}\right)+\mathrm{Size}\left(\mathrm{LT}\right)+\mathrm{Size}\left(\mathrm{ID}\right)+2\times \mathrm{Size}\left({\mathrm{IDB}}_{\mathrm{Sig}}\right). \end{array}$$

To establish the route of the proposed method, the total number of bytes in the packets flowing in the network is expressed as

(23) $$\begin{array}{cc} \; & B_{\mathrm{IR}}=B_{\mathrm{AODV}}-2\left(N+1\right)\times \mathrm{Size}\left(\mathrm{HC}\right)+2\left(N+1\right)\mathrm{Size}\left(\mathrm{LT}\right)+2\left(N+1\right)\times \mathrm{Size}\left(\mathrm{LI}\right)\\ \; & +2N\times \mathrm{Size}\left(\mathrm{ID}\right)+2\left(2N+1\right)\times \mathrm{Size}\left({\mathrm{IDB}}_{\mathrm{Sig}}\right)+\left(N+1\right)L_{\mathrm{DR}}, \end{array}$$

where *N* denotes the number of nodes between the source node S and the responder generating the RREP.

**Figure 3:**
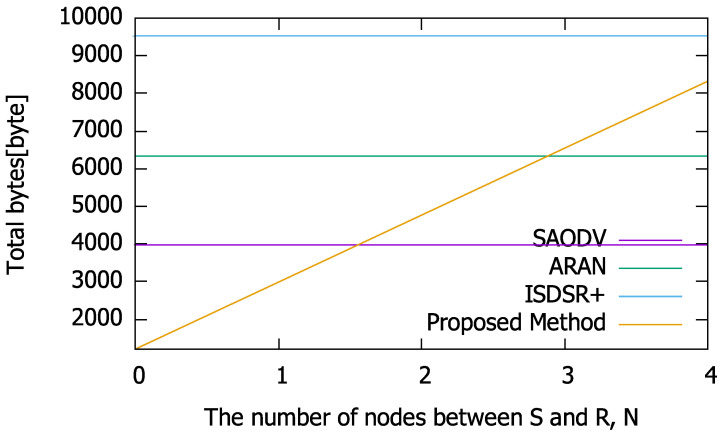
Comparison of the total byte number characteristics with each method at n=5.

As indicated in Equation (23), when the intermediate nodes start to generate RREPs using the proposed method, the total number of bytes always increases with respect to the number of hops as compared to the other methods, which perform end-to-end communication with the same number of hops. This is because the packet size of a single message increases due to the previous packet being written in the RREP. However, in the proposed method the intermediate nodes can generate RREPs, making it possible to reduce the total packet size flowing in the entire network at the time of route construction. This is particularly applicable to large-scale end-to-end networks. By substituting the packet size values shown in Table 1 in Equations (18)–(23), the sum of the packet size with respect to the number of hops can be calculated. The results are presented in Figure 3.

**Figure 4:**
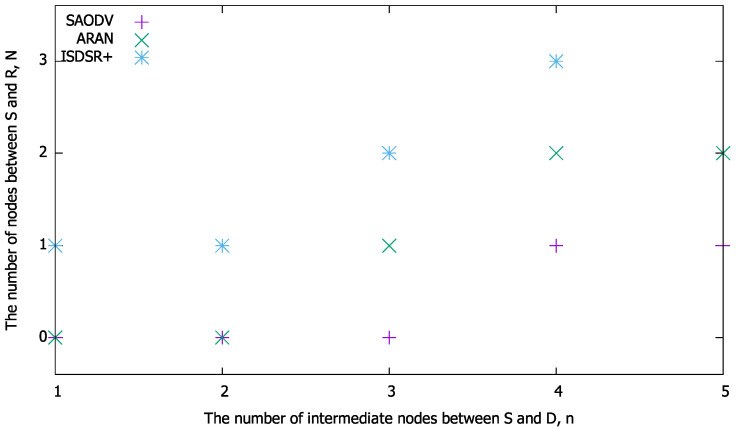
Threshold of *N* for *n*, showing that the proposed method is superior to the existing method in terms of the total bytes.

As shown in Figure 3, when n=5 (i.e., the number of intermediate nodes between the source and destination is 5), it takes twelve hops for successful route construction in the comparison methods. In comparison with ARAN, if the second node or less from the source has a route to the destination and the RREP is generated as a responder, the route can be constructed more efficiently. In contrast, compared to SAODV, the routes can be constructed within four hops using the proposed method. Moreover, our proposed method is always more efficient than ISDSR+ because the signature size of IBSAS is larger than that of the ID-based signature. These results imply that the proposed method has a condition for *N* wherein its communication efficiency is superior to that of existing methods in terms of the total number of bytes. As the total number of bytes depends on *n* (and *N* in the proposed method), *n* affects the condition under which the proposed method is superior to the compared methods. Based on this fact, we calculated (a) the condition of *N* for $B_{\mathrm{IR}}\leq B_{\mathrm{SAODV}}$, (b) the condition of *N* for $B_{\mathrm{IR}}\leq B_{\mathrm{IR}}$, and (c) the condition of *N* for $B_{\mathrm{IR}}\leq B_{\mathrm{ISDSR}}+$. Figure 4 summarizes the maximum value of *N* at which the total number of bytes of the proposed method is smaller than that of the compared methods; these values were obtained based on Equations (18)–(23) and Table 1.

Figure 4 demonstrates the maximum value of *N* at which the proposed method is superior to the compared methods under various network sizes. As evidenced by Figure 4, the region of *N* in which the proposed method is superior widens as *n* increases for each method, that is, the proposed method tends to improve communication efficiency further as the network size increases.

### 6.4. Emulation-Based Analysis for IoT Networks

We have shown that the proposed method can improve communication efficiency while maintaining security performance. However, digital signatures tend to increase the computational load on the device because they rely on public key-based techniques. Thus, evaluating the sum of the communication and computation times to obtain the practical time required for routing is important.

Motivated by this background, we performed an emulation-based routing overhead analysis assuming multi-hop IoT networks (i.e., low-rate communication with limited computational capability).

#### 6.4.1. Emulation Configuration

Similar to the system model illustrated in Figure 1, this emulation assumes a situation where *n* intermediate nodes exist between the source and destination nodes. Each node comprises a Raspberry Pi 4 Model B, which is a low-cost single-board computer. All encryption algorithms were implemented using C language with the GCC compiler, and the calculation time was obtained from the actual runtime on the Raspberry Pi. To evaluate the computational performance, it is necessary to obtain the following computational times: (i) ID-based signature (proposed method and ARAN); (ii) IBSAS (ISDSR+), and (iii) SHA-256 hash chain generation (SAODV). (Note that as the original ARAN utilizes public key-based authentication, which requires a public key infrastructure (PKI), we used the same ID-based signature for the proposed protocol and ARAN here in order to align the evaluation conditions.) All methods were implemented using PBC Library 0.5.14 [31], and the ID-based signature followed the method proposed in [20]. Furthermore, it is assumed that packets are transmitted based on ZigBee-based physical layer specification [32]. The communication component is derived from the theoretical performance of ZigBee-based communication. The main specifications of this emulation are summarized in Table 2.

Based on the above conditions, we evaluated the routing overhead during one routing process by repeatedly generating, verifying, and hashing packets as many times as required by the routing protocols in an environment with *n* relay nodes on a single Raspberry Pi. After this routing emulation had been repeated 1000 times, we calculated the average performance. Note that IDs are assumed to be distributed to nodes in advance. Thus, this emulation result assumes no access points or centralized server (e.g., public key infrastructure (PKI)). In addition, this emulation does not consider any other time, as these times are sufficiently short compared with the communication and cryptographic parts.

#### 6.4.2. Calculation Time

**Figure 5:**
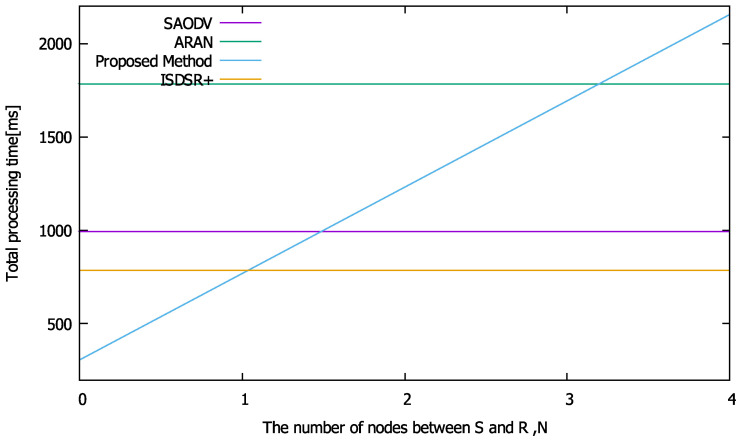
Calculation time performances when n=5.

First, the calculation time required for each cryptographic calculation was evaluated. The average calculation time required for routing is shown in Figure 5. The performances of the compared methods are flat regardless of the *x*-axis because these methods need to communicate via end-to-end routing packets. We repeated the cryptographic operations required for routing in each method 1000 times on a Raspberry Pi and averaged the results. Regarding the communication time (Figure 3), the proposed method can reduce the computation time, as the nodes close to S generate RREPs (i.e., *N* is smaller). However, as the number of hops increases, the computation time increases compared to the other methods, as the proposed method verifies the signatures in past packets, thereby increasing the number of calculations required of each node.

**Figure 6:**
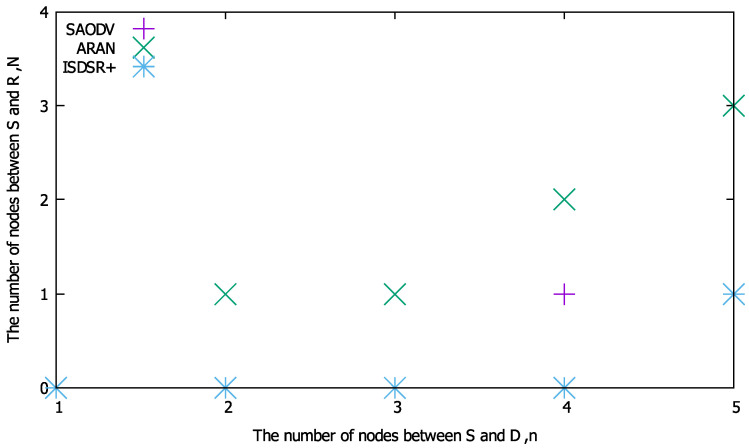
Threshold of *N* for *n* at which the proposed method is superior to the compared methods in terms of calculation time.

Next, the calculation time performance for various numbers of intermediate nodes was evaluated. To clarify whether the proposed method outperforms the others, we evaluated a threshold of *N* where the proposed method is superior to each comparison method in terms of the calculation time. Figure 6 shows the threshold of *N* against the total number of intermediate nodes *n*. This figure reveals that the proposed method can be superior in terms of computation time even for small networks such as n=1,2. As *n* increases the computation time can be further improved, even if a node far from S generates RREPs. In contrast, compared with ISDSR+, the threshold value of *N* is zero even at the scale of n=4, indicating that the conditions for reducing the computation time are limited. This is because ISDSR+ uses IBSAS, which aggregates multiple signatures into a single signature, making it faster than ordinary ID-based signatures.

#### 6.4.3. Routing Overhead

Here, we present a comparison of the total routing time. This performance can be obtained by summing the calculation time for cryptographic processing (obtained in Section 6.4.2) and the communication time for relaying the routing packets.

Let us consider a situation in which a packet is transmitted from one node to another node with a transmission rate *R* (in bps) and the receiver is within the transmitter’s communication coverage. If routing is performed by *n* end-to-end nodes, the communication time can be derived as

(24) $$t_{\mathrm{com}}=\sum_{i=1}^{n+1}\frac{M_{\mathrm{req},i}}{R}+\sum_{j=1}^{n+1}\frac{M_{\mathrm{rep},j}}{R}\;\;\left[s\right].$$

where $M_{\mathrm{req},i}$ is the packet length (bits) in the *i*-th hop of the RREQ phase (counted from the source node) and $M_{\mathrm{rep},j}$ is the packet length (bits) in the *j*-th hop of the RREQ phase (counted from the destination node). Similarly, the performance of the proposed method when an intermediate node initiates the RREP can be derived by substituting *N* into *n* in Equation (24).

Based on the ZigBee specification [32], we assume $R=250\times 10^{3}$ (bps). When the communication time is obtained, we can derive the routing overhead by summing it with the calculation time obtained from Figure 5.

**Figure 7:**
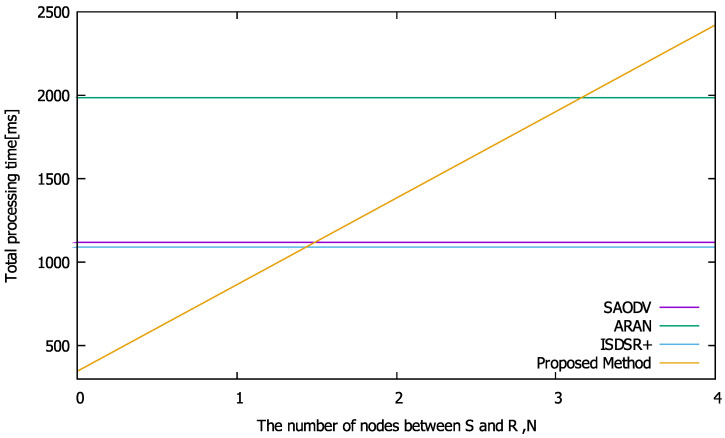
Routing overhead performance where n=5.

**Figure 8:**
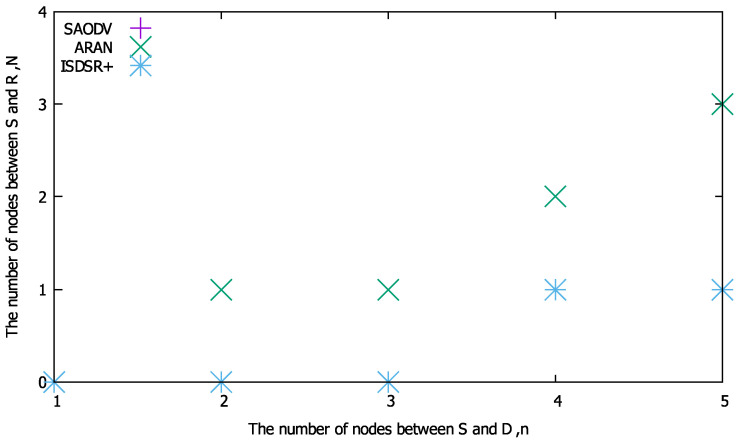
Threshold of *N* for *n* at which the proposed method is superior to the compared methods in terms of the routing overhead.

Figure 7 shows the overhead performance, where n=5. If N=0 or 1 (i.e., the proposed method can construct the route in four hops), our proposed method can reduce the route construction time relative to the compared methods. Next, we illustrate the maximum number of nodes between S and the replying node *N* at which the proposed method is superior to the comparison methods in terms of routing overhead in Figure 8. This figure reveals that as the network becomes larger (i.e., *n* increases), our proposed method is better able to reduce the construction time. However, compared with SAODV and ISDSR+, the conditions for reducing the route construction time are limited (e.g., N=0 at n=3). Although the proposed method can reduce the required number of communications for route construction, the cryptographic computation time becomes a bottleneck, as shown in Figure 5. However, as discussed in Section 6.3, the proposed method outperforms all of the compared methods in terms of the total number of bytes.

### 6.5. Emulation-Based Analysis under Dynamic Topology

In practice, the routing performance is affected by topology dynamics owing to various uncertainties, including mobility and interference. To evaluate these effects, we performed numerical simulations of routing protocols assuming distributed and dynamic wireless networks. Specifically, in order to evaluate the uncertainties in communications this simulation assumes routing on distributed sensor networks in which a routing packet cannot be decoded with an outage probability $p_{\mathrm{out}}$.

**Figure 9:**
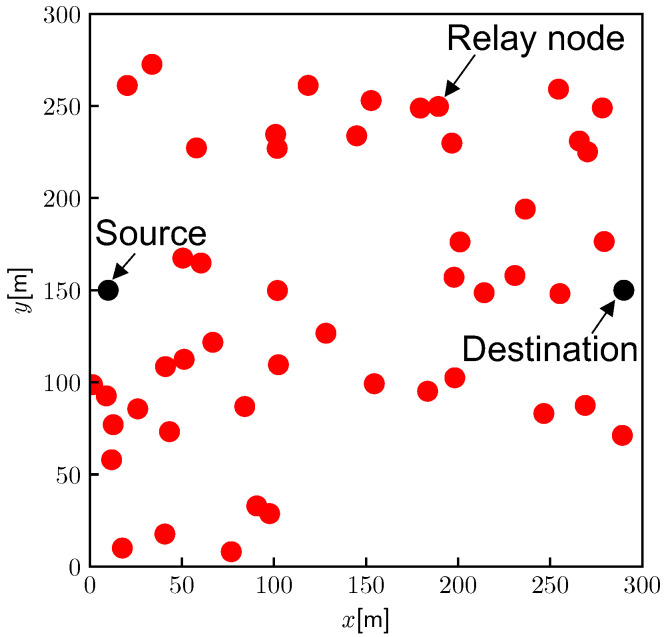
Node deployment example.

This simulation assumes that $n_{\mathrm{relay}}$ nodes are randomly deployed in a 300 square meter area, as shown in Figure 9. The source and destination are fixed at (10 m, 150 m) and (290 m, 150 m), respectively. The nodes have communication coverage $d_{\max}$ [m], and a broadcast signal can be decoded when the communication distance is below $d_{\max}$; however, it cannot be decoded with an outage probability $p_{\mathrm{out}}$ even within the coverage. Node locations are assumed to be fixed during the routing process because they are able to finish within 1–2 s.

**Figure 10:**
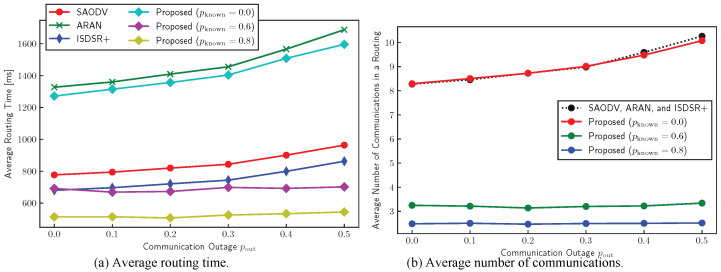
Effects of outage probability on average routing time (30 relay nodes).

Figure 10a shows the effects of $p_{\mathrm{out}}$ on the average routing time. We iterated this simulation 1000 times with different node deployments and evaluated the average performance. The *y*-axis indicates the sum of the communication and computation times. In addition, the computation times were measured on a Raspberry Pi, as with the emulation-based analysis in Section 6.4. Furthermore, we introduced the *route known probability*, $p_{\mathrm{known}}$, for the relay nodes where a relay node knows the route to the destination. With the proposed protocol, a relay node receiving a route request (RREQ) can begin to return the route reply (RREP) if it knows a route to the destination. However, SAODV, ARAN, and ISDSR+ allow nodes to perform end-to-end communication at any time.

This figure shows that when the chance of a communication outage increases the routing time, the proposed method remains robust to outage events, specifically at a high $p_{\mathrm{known}}$. This is because the proposed method can avoid end-to-end routing if a relay node knows a route to the destination. Figure 10b illustrates the average number of communication times during routing. The proposed method is able to reduce the required number of communication times.

**Figure 11:**
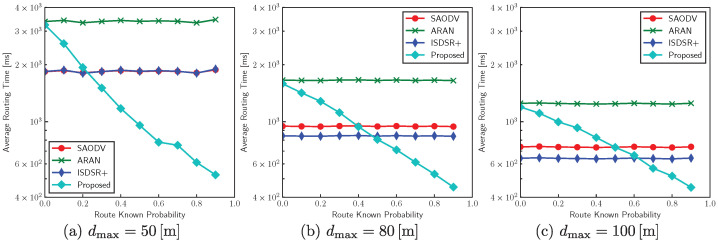
Effects of route known probability on the average routing time (50 relay nodes and $p_{\mathrm{out}}=0$).

The effects of communication distance $d_{\max}$ on the average routing time are shown in Figure 11. We performed simulations with $d_{\max}=50,80,\;\mathrm{and}\;100$ (in m) with various $p_{\mathrm{known}}$. The proposed method is advantageous when $p_{\mathrm{kown}}$ is high. In practice, $p_{\mathrm{known}}$ tends to be higher as the number of communications increases. In addition, the proposed method can achieve faster routing in environments where the communication distance is short; for example, the proposed protocol can improve the average routing time more than three-fold compared with conventional methods when $d_{\max}=50$ and $p_{\mathrm{known}}=0.90$. This is because the compared methods require a large number of hops for end-to-end communications. In contrast, the proposed method can significantly improve the routing characteristics by reducing the number of hops.

### 6.6. Discussion

Having performed theoretical and emulation-based analyses in terms of routing overhead, we now discuss application-specific scheme design based on our evaluation results.

The proposed method has lower latency when the communication distance is shorter, and the route known probability $p_{\mathrm{known}}$ increases. For example, Figure 11a shows that with $d_{\max}=50$ (m) and $p_{\mathrm{known}}=0.8$ it is more than three times faster than other methods. As $p_{\mathrm{known}}$ increases with the number of previous routing attempts, the proposed protocol is suitable for applications that require low latency, short communication distances, and static terminal locations, such as IoT environmental monitoring and decentralized edge learning in urban situations. A similar trend is expected for long communication distances over a wide area. Therefore, it is suitable for wide-area communication using a low-power wide-area (LPWA) network as well.

However, when $p_{\mathrm{known}}$ is low, the proposed method requires end-to-end communication in many cases. Unlike the compared methods, the proposed method increases the packet length hop-by-hop. For environments in which terminals move rapidly, such as vehicular networks, it is preferable to choose other schemes, such as ISDSR+.

## 7. Conclusions

To improve the communication efficiency of cryptography-based secure routing, we have proposed a secure routing protocol that enables the relay node to initiate an RREP. Our proposed method enables an intermediate node to prove to a third party that it has a possible route to the destination by disclosing the previous route information received from the destination, significantly reducing the number of hops required for route establishment compared with existing methods. Furthermore, we have performed an emulation-based routing overhead analysis assuming multi-hop IoT networks. The emulation results demonstrate that the proposed method improves communication efficiency in IoT systems that operate in environments where communication failure due to signal interference is a concern, such as congested ISM bands.

Finally, we summarize the limitations of the proposed method and discuss future works. The proposed method increases the packet length hop-by-hop, which degrades the routing overhead in end-to-end communication. Introducing efficient authentication schemes, such as physical layer authentication [34], is a possible future direction. Furthermore, the routing performance degrades under heterogeneous computing and communication capabilities. Because this heterogeneity sometimes behaves as an attack on routing, improving these effects should be discussed as a next step. Finally, the integrated design of low-latency routing and novel applications in distributed networks is a possible direction. For example, recent demands for big data analysis have attracted considerable attention for FL [6]. FL is expected to be a privacy-preserving machine learning scheme, and can extend its training scale based on multi-hop communications; however, it should be performed with high security and communication efficiency. Thus, the design of routing for FL over large-scale wireless networks represents an interesting research topic. 

## Figures and Tables

**Table 1:** Parameters for performance evaluations.

Contains	Size [Byte]
$\mathrm{Size}({\mathrm{RREQ}}_{\mathrm{AODV}})$	24
$\mathrm{Size}({\mathrm{RREP}}_{\mathrm{AODV}})$	20
$\mathrm{Size}({\mathrm{RREQ}}_{\mathrm{DSR}})$	$\sum_{k=1}^{n+1}\left(12+4\left(k-1\right)\right)$
$\mathrm{Size}({\mathrm{RREP}}_{\mathrm{DSR}})$	12+4(k−1)
Size(ID)	4
Size(HC)	1
Size(LT)	8
Size(LI)	8
Size(Hash)	32
$\mathrm{Size}({\mathrm{IDB}}_{\mathrm{Sig}})$	276
$\mathrm{Size}({\mathrm{IBSAS}}_{\mathrm{Sig}})$	768
Size(Nonce)	5

**Table 2:** Emulation specifications.

Implementation Device	Raspberry Pi 4 (Model B)
OS	Ubuntu 18.04 LTS
CPU	1.5-GHz Quad-Core Cortex-A72
Main memory	LPDDR4-2400 SDRAM 4 GB
Language	C with GCC 7.5.0
External libraries	OpenSSL 1.1.1d [33] and PBC Library 0.5.14 [31]
Transmission rate *R*	250 [kbps]

## Data Availability

Not applicable.
